# Tip110/SART3-Mediated Regulation of NF-κB Activity by Targeting IκBα Stability Through USP15

**DOI:** 10.3389/fonc.2022.843157

**Published:** 2022-04-21

**Authors:** Khalid Amine Timani, Sahar Rezaei, Amanda Whitmill, Ying Liu, Johnny J. He

**Affiliations:** ^1^ Department of Microbiology and Immunology, Rosalind Franklin University, Chicago Medical School, North Chicago, IL, United States; ^2^ Center for Cancer Cell Biology, Immunology and Infection, Rosalind Franklin University, North Chicago, IL, United States; ^3^ School of Graduate and Postdoctoral Studies, Rosalind Franklin University, North Chicago, IL, United States; ^4^ Department of Microbiology, Immunology, and Genetics, University of North Texas Health Science Center, Fort Worth, TX, United States

**Keywords:** Tip110/SART3, NF-kB, IkB kinase, USP15, inflammation

## Abstract

To date, there are a small number of nuclear-restricted proteins that have been reported to play a role in NF-κB signaling. However, the exact molecular mechanisms are not fully understood. Tip110 is a nuclear protein that has been implicated in multiple biological processes. In a previous study, we have shown that Tip110 interacts with oncogenic ubiquitin specific peptidase 15 (USP15) and that ectopic expression of Tip110 leads to re-distribution of USP15 from the cytoplasm to the nucleus. USP15 is known to regulate NF-κB activity through several mechanisms including modulation of IκBα ubiquitination. These findings prompted us to investigate the role of Tip110 in the NF-κB signaling pathway. We showed that Tip110 regulates NF-κB activity. The expression of Tip110 potentiated TNF-α-induced NF-κB activity and deletion of the nuclear localization domain in Tip110 abrogated this potentiation activity. We then demonstrated that Tip110 altered IκBα phosphorylation and stability in the presence of TNF-α. Moreover, we found that Tip110 and USP15 opposingly regulated NF-κB activity by targeting IκBα protein stability. We further showed that Tip110 altered the expression of NF-κB-dependent proinflammatory cytokines. Lastly, by using whole-transcriptome analysis of Tip110 knockout mouse embryonic stem cells, we found several NF-κB and NF-κB-related pathways were dysregulated. Taken together, these findings add to the nuclear regulation of NF-κB activity by Tip110 through IκBα stabilization and provide new evidence to support the role of Tip110 in controlling cellular processes such as cancers that involve proinflammatory responses.

## Background

Nuclear factor of κB (NF-κB) is a critical mediator of the cellular response to inflammatory cytokines, developmental signals, pathogens, and cellular stress in which its constitutive activation promotes tumor initiation and development (1, 2). NF-κB is activated by a mystifying array of stimuli, including biological agents such as tumor necrosis factor α (TNF-α), interleukin-1, bacterial endotoxin, and phorbol esters and cytotoxic stimuli such as chemotherapeutic agents and oxidative stress (3). The activity of NF-κB is regulated through its interaction with inhibitor proteins (IκBs), which prevent DNA binding to the p65 subunit of NF-κB. The NF-κB/IκB complex is localized exclusively in the cytosol because of a nuclear export signal encoded in the IκB subunit and the masking of a nuclear localization signal (NLS) in the NF-κB subunit (4). Proinflammatory stimuli activate IκB kinase (IKK) to phosphorylate IκBα at two N-terminal serines, Ser32 and Ser36. This phosphorylation triggers ubiquitination at the N-terminal lysines, Lys20 and Lys21 in IκBα, leading to its degradation through the 26S proteasomal pathway and subsequent translocation of p65 to the nucleus, where it regulates transcription of target genes (5–7). Thus, identifying the molecular players that regulate NF-κB and characterization of the mechanistic pathways through which the molecular players affect NF-κB activation will provide clues for possible therapeutic strategies against inflammatory diseases and cancer.

HIV-1 Tat-interacting protein 110 (Tip110), also known as “squamous cell carcinoma antigen recognized by T cells 3” (SART3), is a nuclear protein that plays an important role in pre-mRNA splicing, spliceosome assembly, and embryonic development (8–15). Tip110 protein expression is very low in normal tissues and non-proliferating cells but becomes highly elevated in a number of malignant tumor cell lines and cancerous tissues as well as in stem cells. Tip110 has been proposed as a potential antigen for cancer immunotherapy (8, 16–19), and its mutations have been linked to inflammatory skin diseases (20). We have summarized the biological functions of Tip110 in a comprehensive review (8). Furthermore, we reported that exposure of a highly metastatic melanoma cell line to severe hypoxic conditions led to significant down-regulation of Tip110 both *in vitro* and *in vivo* (21). Also, we have shown that Tip110 regulates interleukin-8 (IL-8) expression and predicts the clinical outcomes in melanoma, indicating that Tip110 expression level could play a role in melanoma tumor progression (22). Furthermore, we and others reported that Tip110 interacts with and/or regulates several oncogenic proteins such as C-Myc, Y-box-binding protein 1 (YB-1), p53, ubiquitin specific peptidase 15 (USP15), and ubiquitin specific peptidase 4 (USP4) (9, 23–25). Interestingly, these Tip110-interacting partners play roles in NF-κB signaling through interaction or regulation of the signaling components of the NF-κB pathway (9, 10, 21, 24, 26, 27). Given the importance of NF-κB signaling in inflammation, immunity, and cell fate decision, we examined the possible role of Tip110 in the regulation of the NF-κB pathway.

In this study, we showed that Tip110 expression regulates TNF-α-induced NF-κB activation, and the nuclear localization signal on Tip110 is required for this activation. Then we explored the potential mechanism underlying the Tip110-mediated NF-κB activity by studying its effects on IκBα protein stability. Then we determined the role of Tip110-associated protein USP15, whose nuclear localization is affected by Tip110 expression, on Tip110-potentiated TNF-α-induced NF-κB activity. Finally, dysregulation of NF-κB and NF-κB-related signaling pathways by Tip110 was further substantiated by using whole-transcriptome analysis of Tip110 knockout cells.

## Results

### Tip110 Activated NF-κB Transcriptional Activity

**Figure 1:**
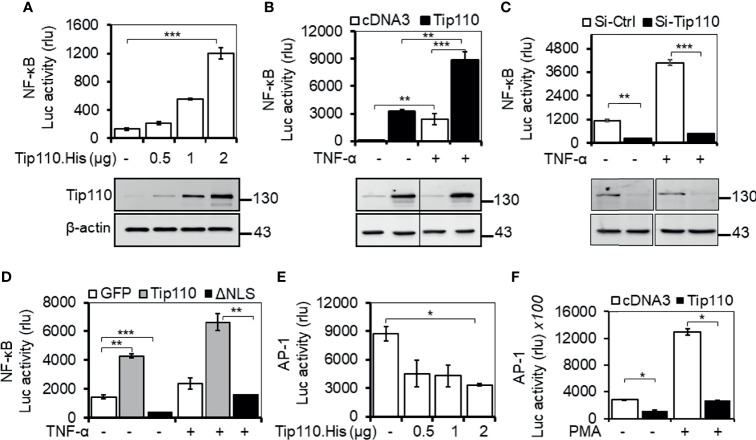
Impact of Tip110 on NF-κB transcriptional activity. **(A-D)**. 293T were transfected with 0.06 µg pGL3-NF-κB(3)-luc and an increasing amount of Tip110. His **(A),** 2.5 µg Tip110.His **(B),** 50 nM Tip110-specific siRNA (si-Tip110) **(C)**, or 2 µg pGFP.Tip110 or pGFP.Tip110ΔNLS containing deletion of the nuclear localization signal (NLS) **(D)**. **(E, F)** 293T were transfected with 0.04 µg pAP-1-Luc and an increasing amount of Tip110.His **(E)** or 2.5 µg Tip110.His **(F)**. pcDNA3 **(A, B, E, F)**, control siRNA (Si-Ctrl, **C**), pGFP **(D)** was used to equalize the total amount of DNA or siRNA among the transfection, and pGFP was added to ensure comparable transfection efficiencies among all transfections. **(B–D)**, 10 ng/ml TNF-α **(F)** 100 ng/ml PMA were added 24 hr post-transfection and continued for either 18 or 6 hr, respectively. Cells were then harvested for the luciferase reporter gene assay, and Western blotting was used to determine the expression of Tip110. β-actin was used as an internal loading control **(A–C)**. The data were means ± SE from triplet samples and representative of three independent experiments. *P < 0.05, **P < 0.01, ***P < 0.001.

**Figure 2:**
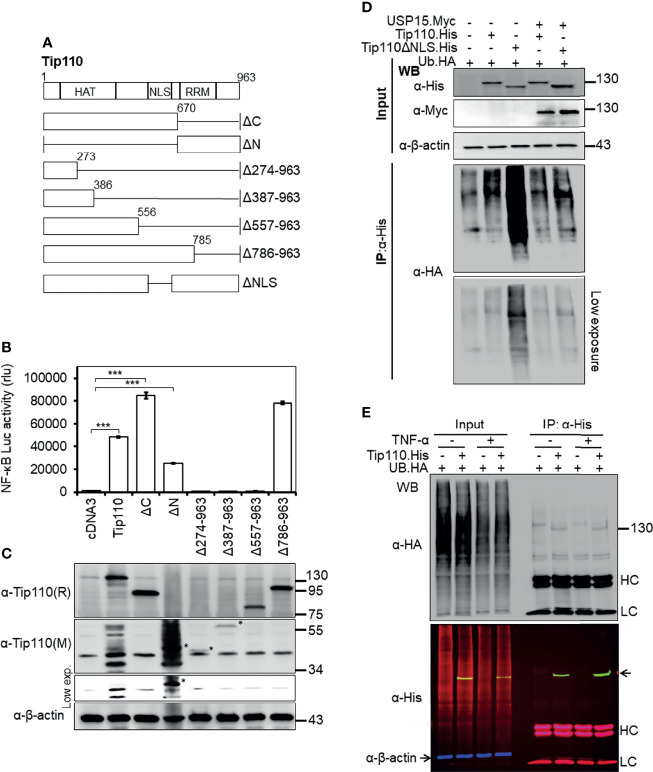
Effects of Tip110 intracellular localization and ubiquitination on NF-κB activity. **(A)** Schematic of Tip110 and its respective deletion mutants. Tip110 consists of seven half-a-tetratricopeptide repeats (HAT), a nuclear localization signal (NLS), and two RNA recognition motifs (RRM). **(B, C)** 293T were transfected with 0.6 µg pGL3-NF-κB(3)-Luc and Tip110.His or one of the Tip110 deletion mutants. Cells were harvested 48 hr post-transfection for the luciferase reporter gene assay **(B)** and Western blotting for Tip110 and its mutant expression using a monoclonal (M) and polyclonal (R) anti-Tip110 antibody **(C)**. **(D)** 293T were transfected with UB.HA and Tip110.His, or Tip110ΔNLS, with and without USP15.Myc cultured for 48 hr, treated with MG132 (10 µM) for 20 hr, and then harvested for Tip110 expression by Western blotting using anti-His antibody (Input) or immunoprecipitated using an anti-HA antibody, followed by Western blotting using anti-HA antibody after the SDS-PAGE was run for a long time to capture polyubiquitinated smear above the Tip110 band. **(E)** 293T were transfected with UB.HA and Tip110.His, cultured for 24 hr, treated with TNF-α (10 ng/ml) for 30 min, and then harvested for expression of total ubiquitin, Tip110, and β-actin by Western blotting using anti-HA and anti-His antibody (Input) or immunoprecipitated using anti-His antibody followed by the same Western blotting to identify the amount of the ubiquitinated Tip110. An Alexa Fluor 488 (green), 555 (red), and 633 (purple) secondary antibodies were used to detect the Tip110, UB, and β-actin, respectively. Tip110 bands in the input appeared in yellow due to the overlay of green (Tip110) and red (ubiquitin) fluorescence. pcDNA3 was used to equalize the total amount of DNA among the transfection, and pGFP expression vector was added to ensure comparable transfection efficiencies among all transfections. β-actin was used as an internal loading control. The data were means ± SE from triplet samples and representative of three independent experiments. HC, IgG heavy; LC, IgG light chain. ***P < 0.001.

Tip110 is identified as an interacting partner with HIV-1 Tat protein that has been reported to enhance NF-κB activity (28, 29). USP15, another Tip110-interacting protein, that has been reported to regulate NF-κB activity (27, 30–32). In addition, Tip110 interacts with and/or regulates other proteins which are known to play a role on NF-κB by targeting NF-κB signaling components (9, 10, 21, 24, 26, 27). All those findings prompted us to investigate the possible role of Tip110 in the NF-κB signaling pathway. Transfection of luciferase-reporter vector pGL3-NF-κB(3)-Luc containing NF-κB responsive element with an increasing amount of Tip110 in 293T enhanced NF-κB transcriptional activity in a dose-dependent manner (**Figure 1A**). Treatment of Tip110 expressing cells with TNF-α further augmented NF-κB transcriptional activity compared to untreated cells (**Figure 1B**). On the contrary, Tip110 knockdown by siRNA in 293T led to inhibition in the NF-κB transcriptional activity in response to TNF-α treatment (**Figure 1C**). Interestingly, deletion of Tip110 nuclear localization signal (NLS) (**Figure 2A**) abolished the NF-κB transcriptional activity (**Figure 1D**).

Even though NF-κB and AP-1 transcription factors are regulated by different mechanisms, they appear to be activated by the same multitude of stimuli (33–35). Indeed, the activation of JNK by inflammatory cytokines or by stress is often accompanied by the nuclear translocation of NF-κB, and many genes require the concomitant activation of AP-1 and NF-κB, suggesting that these transcription factors work cooperatively (36, 37). To determine whether Tip110 expression would also regulate AP-1 transcriptional activity, 293T were transfected with a luciferase-reporter vector containing the AP-1 responsive element with an increasing amount of Tip110. The results showed that Tip110 expression suppressed AP-1 transcriptional activity in a dose-dependent manner (**Figure 1E**). A similar result was also found under phorbol 12-myristate 13-acetate (PMA) treatment (**Figure 1F**). Under our experimental settings, TNF-α showed low activation of AP-1 luciferase activity in 293T, and the effect was not significant; therefore, we used PMA, a known activator for AP-1 transcriptional activity. These data suggest that Tip110 activates NF-κB transcriptional activity through Tip110 nuclear localization but inhibits AP-1 transcriptional activity.

### N-Terminal Domain on Tip110 Responsible for the NF-κB Activation

Tip110 contains 12 half-a-tetratricopeptide repeats (HAT) and an NLS at the N terminus and two RNA recognition motifs (RRM) at the C terminus. To identify the Tip110 domains involved in the regulation of NF-κB activity, a series of Tip110 mutant proteins (**Figure 2A** **(** 25, 28 **),** were analyzed. Similarly, 293T were transfected with wild-type Tip110 and its mutants together with the NF-κB reporter. Compared to wild-type Tip110, deletion of Tip110 N-terminal domain (Tip110ΔN), including the NLS, resulted in a decrease in the NF-κB activity (**Figure 2B**). Deletion up to 557 aa from the C-terminal, including the NLS, completely abolished NF-κB activity similar to the cDNA3-transfected control. While deletion of the C-terminal domain on Tip110 (Tip110ΔC and Tip110Δ785-963 mutants) led to higher NF-κB activity than the wild-type (**Figure 2B**). Western blotting was performed to confirm the expression of Tip110 mutant proteins (**Figure 2C**). These results demonstrated that an approximately 300 aa at the N-terminal domain, which includes the NLS region on Tip110, 556-785 aa, is required for the NF-κB activation.

We have previously shown that Tip110 is ubiquitinated and this ubiquitination is regulated by its associated-protein USP15 (25). Therefore, we then determined whether Tip110 ubiquitination would be affected by its intracellular localization. 293T were transfected with UB.HA alone or plus Tip110.His or Tip110ΔNLS.His on the presence or absence of pUSP15.Myc. Cells were treated with MG132, an inhibitor of 26S proteasome degradation, then collected for cell lysates. Tip110 expression was confirmed by Western blotting (**Figure 2D**, Input). Immunoprecipitation using anti-His antibody followed by Western blotting using anti-HA antibody led to the detection of a highly ubiquitinated Tip110ΔNLS mutant protein compared to wild-type Tip110 and the expression of USP15 deubiquitinated both Tip110 and its cytoplasmic mutant Tip110ΔNLS (**Figure 2D**, IP). Then we used the same strategy to determine whether TNF-α stimulation would alter Tip110 ubiquitination. 293T were transfected with Tip110.His or its control plasmid then either treated with TNF-α or left untreated. Immunoprecipitation assay data showed little effect in ubiquitinated Tip110 upon TNF-α stimulation compared to unstimulated cells with reference to the amount of Tip110 input (**Figure 2E**). Those results indicate that Tip110 ubiquitination level affects its intracellular localization and might be involved in NF-κB activity.

### Tip110 Promoted TNF-α-Induced Nuclear Translocation of NF-κB p65

**Figure 3:**
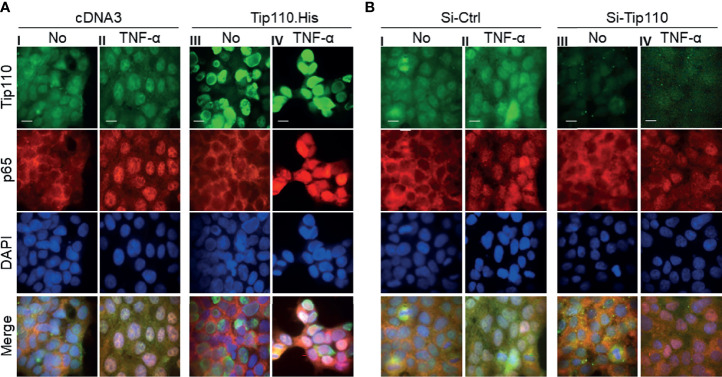
Effects of Tip110 expression and TNF-α treatment on NF-κB p65 nuclear translocation. **(A)** 293T were transfected with 0.8 µg Tip110.His **(A)** 50 nM Si-Tip110 **(B)**, cultured for 24 hr, treated with 20 ng/ml TNF-α for 30 min, and then processed for immunostaining using anti-Tip110 and anti-p65 antibodies. The cells were counterstained with DAPI for nuclei. pcDNA3 or si-Ctrl was used as the respective controls. Representative micrographs were separately taken using fluorescence microscopy and used to make the composite (Merge). Magnification; 100X, Scale bar, 10µM.

We and others have shown that a number of Tip110-associated proteins translocated to the nucleus when they co-expressed with Tip110 such as USP15, USP4, and YB-1 ((12, 25, 38), data not shown for YB-1). In addition, NF-κB activation is mainly associated with nuclear translocation of endogenous NF-κB p65 (2). Thus we analyzed the distribution of NF-κB p65 between the cytoplasm and nucleus in relation to Tip110 expression and TNF-α treatment by using an immunofluorescence assay. Expression of endogenous NF-κB p65 localized mainly in the cytoplasm and weak expression was also detected in the nucleus due to its continuous shuttling between both compartments (39) (**Figure 3A–I**). Treatment of cells with TNF-α for 30 min caused accumulation of NF-κB p65 in the nucleus (**Figure 3A–II**). Tip110 overexpression showed no significant effect on p65 nuclear accumulation (**Figure 3A–III**). However, treatment of Tip110 expressing cells with TNF-α greatly enhanced NF-κB p65 nuclear accumulation compared to control expressing cells (**Figure 3A–IV**). On the other hand, Tip110 knockdown by siRNA in the presence or absence of TNF-α treatment has no significant effect on NF-κB p65 protein expression and localization pattern compared to si-RNA control cells (**Figures 3B–III, IV**). Then we determined whether Tip110 would be associated with NF-κB p65. 293T were transfected with Tip110 or vector control then treated with TNF-α for 20 hr. Cells were lysed, and an immunoprecipitation assay was performed. Tip110 and p65 were expressed (**Figure S1A**), but there was no complex formation between Tip110 and p65 (**Figure S1B** **)**. These data together demonstrated that Tip110 expression altered NF-κB p65 nuclear accumulation only in the presence of TNF-α stimulation, suggesting that other mechanisms may also contribute to NF-κB activation by Tip110.

### Tip110 Altered IκBα Phosphorylation and Stability

**Figure 4:**
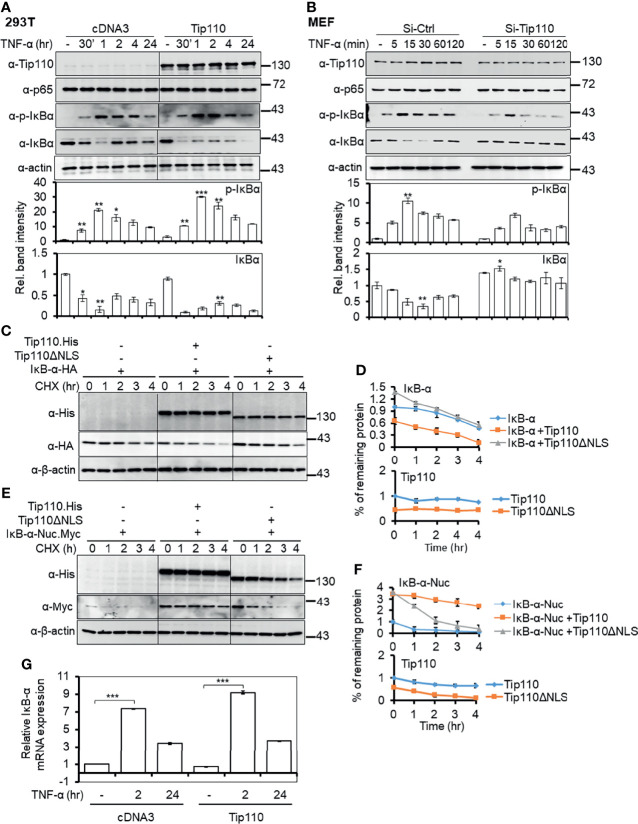
Effects of Tip110 expression on IκBα phosphorylation and protein stability. **(A)** 293T were transfected with Tip110.His and its plasmid control, treated with 20 ng/ml of TNF-α for 0, 30 min, 1, 2 4 and 24 hr then harvested for Western blotting. pcDNA3 was used to equalize the total amount of DNA among the transfection. **(B)** Primary mouse embryonic fibroblasts (MEF) were transfected with 50 nM Si-Ctrl or Si-Tip110, cultured for 24 hr, treated with 20 ng/ml TNF-α for 0, 5, 15, 30, 60, or 120 min. 293T were transfected with **(C)** IκBα.HA or **(E)** IκBα.Nuc.Myc and Tip110.His or Tip110ΔNLS.His, cultured for 48 hr, treated with 20 µg/ml cycloheximide (CHX) for 0, 1, 2, 3, and 4 hr, and then harvested for Western blotting. Relative IκBα protein level was normalized to β-actin and expressed as a percentage of the untreated or as individual values **(D, F)**. **(G)** 293T were transfected with Tip110 or pcDNA3, cultured for 48 hr, treated with TNF-α (10 ng/ml) for 2 or 24 hr, and harvested for total RNA isolation and qRT-PCR for IκBα mRNA level. Relative IκBα mRNA was normalized to β-actin and calculated using the untreated as a reference. *P < 0.05, **P < 0.01, ***P < 0.001.

The canonical NF-κB transactivation pathway involves IκBα phosphorylation and degradation (2). Thus, we next investigated whether Tip110 expression would alter IκBα expression and stability in the presence TNF-α stimulation. 293T were transfected with Tip110 expression vector or its control and treated with TNF-α for various lengths of time. Western blotting showed that under TNF-α stimulation, phosphorylated IκBα increased under the context of Tip110 expression (**Figure 4A**), which is paralleled with changes in the IκBα protein stability. To further investigate whether this effect could also be observed in normal primary cells which retain a native signaling pathways, we used primary mouse embryonic fibroblasts (MEF) under the context of limited amount of Tip110. Cells were transfected with siRNA-Tip110 or control siRNA, treated with TNF-α at various lengths of time up to 120 min, and harvested for Western blotting. Similarly, Tip110 knockdown led to decreases in IκBα phosphorylation and subsequent stabilization of the total amount of IκBα in the presence of TNF-α stimulation (**Figure 4B**). These results also showed that Tip110 had no significant effects on the amount of NF-κB p65 protein. These data suggest that Tip110 alters the phosphorylation status and stability of IκBα and subsequently regulates NF-κB activity under TNF-α stimulation.

Unexpectedly, no significant effect was detected on the basal level of IκBα in the absence of TNF-α treatment (**Figures 4A, B**). Thus to further examine whether Tip110 expression regulates IκBα protein turnover rate, a cycloheximide (CHX) chase assay was performed. 293T were transfected with IκBα.HA alone or plus Tip110.His or Tip110ΔNLS expression vectors, treated with CHX, to inhibit new protein synthesis, for various lengths of time, and harvested to determine Tip110 and IκBα protein expression by Western blotting. Tip110 overexpression accelerated IκBα degradation compared to its transfection control (**Figures 4C, D**), while expression of Tip110ΔNLS that previously showed an inhibitory effect on NF-κB activity (**Figure 1D**) showed no apparent effects on IκBα protein level compared to its transfection control (**Figures 4D, E**). Further, we observed that Tip110ΔNLS expression caused more accumulation of IκBα protein before the beginning of CHX treatment compared to Tip110 and the control (**Figures 4C, E**). Then we examined whether Tip110 or Tip110ΔNLS expressions affect the turnover rate of the nuclear targeted IκBα by using IκBα-Nuc.Myc expression plasmid. Interestingly, CHX chase assay showed that Tip110 expression stabilized the nuclear IκBα compared to its transfection control while the expression of Tip110ΔNLS accelerated nuclear IκBα degradation compared to Tip110 but still higher than the transfection control (**Figures 4E, F**). In addition, qRT-PCR results showed that Tip110 expression only had a little effect on the IκBα mRNA level at 2 hr, but had no effect at 24 hr following TNF-α treatment compared to the control transfection (**Figure 4G**). These data showed that the Tip110 affects IκBα phosphorylation under TNF-α treatment and the regulation of IκBα protein stability.

### Tip110 and USP15 Opposingly Regulated NF-κB Activity by Targeting IκBα

**Figure 5:**
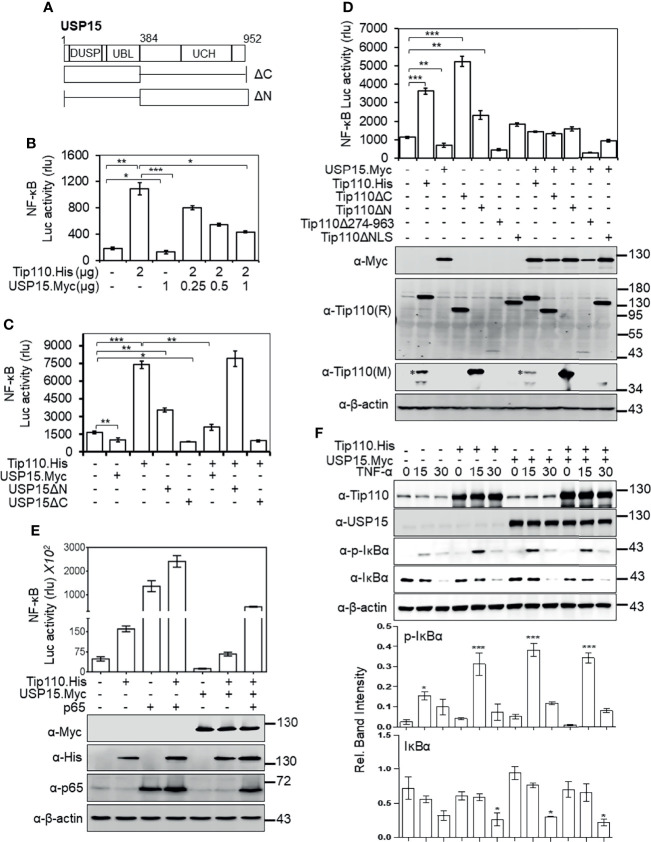
USP15 abrogated Tip110-induced NF-κB activity by targeting IκBα. **(A)** Schematic of USP15 protein and its respective deletion mutants. USP15 contains three distinct domains: domain present in ubiquitin-specific proteases (DUSP), ubiquitin-like domain (UBL), ubiquitin C-terminal hydrolase (UCH). **(B)** 293T were transfected with pGL3-NF-κB(3)-Luc, Tip110.His, and an increasing amount of USP15.Myc, cultured 48 hr, and harvested for the luciferase reporter gene assay. **(C)** 293T were transfected with pGL3-NF-κB(3)-Luc, Tip110.His, USP15.Myc, USP15ΔN, or USP15ΔC, cultured for 48 hr, and harvested for the luciferase reporter gene assay. **(D)** 293T were transfected with pGL3-NF-κB(3)-luc,USP15.Myc, Tip110.His or Tip110 deletion mutants. **(E)** 293T were transfected with pGL3-NF-κB(3)-Luc, Tip110.His, USP15.Myc, and p65. Cells were processed the same as above, and Western blotting using indicated antibodies. pcDNA3 was used to equalize the total amount of DNA among the transfection, and pGFP expression vector was added to ensure comparable transfection efficiencies among all transfections. The data were means ± SE from triplet samples and representative of three independent experiments. **(F)** 293T were transfected with Tip110.His and/or USP15.Myc, treated with 20 ng/ml TNF-α for 15 or 30 min, and harvested for Western blotting using antibodies as indicated. *P < 0.05, **P < 0.01, ***P < 0.001.

We showed that nuclear localization was required for potentiation of TNF-α-induced NF-κB activation by Tip110 and different effect of Tip110 on the IκBα protein stability is dependent on its localization (**Figures 1**, **4**). The TNF-α-induced NF-κB signaling cascade is initiated within the cytoplasm (37). Furthermore, Tip110 interacts with USP15 and regulates its localization between the cytoplasm and nucleus (25, 38), while USP15 is shown to regulate NF-κB activity (27, 40, 41). Those findings together raised the possibility that USP15 might be the link between Tip110 and NF-κB activation. To test this possibility, 293T were transfected with pGL3-NF-κB(3)-Luc, Tip110, USP15, or USP15 mutants (**Figure 5A**). The data showed that the expression of USP15 inhibited NF-κB transcriptional activity (**Figure 5B**). Interestingly, increasing the amount of USP15 expression plasmid in the presence of Tip110 led to gradual decreases of Tip110-induced NF-κB activity in a dose-dependent manner (**Figure 5B**). Then we determined which USP15 domain was involved in the inhibition of NF-κB activity. The results showed that the N-terminal domain of USP15, which contains the domain present in ubiquitin-specific proteases (DUSP) and ubiquitin-like domain (UBL) (**Figure 5A**), was responsible for this inhibition as deletion of this domain (USP15ΔN) enhanced the NF-κB activity (**Figure 5C**). While deletion of the C-terminal domain, USP15ΔC, led to inhibition of NF-κB activity. Furthermore, co-expression of Tip110 with the USP15ΔC mutant led to reduction on the Tip110-induced NF-κB activity. In contrast, co-expression with the USP15ΔN mutant had no such effects (**Figure 5C**), indicating that the N-terminal domain of USP15, which physically associates with Tip110 (25), is responsible for USP15 inhibitory function on Tip110-induced NF-κB activity.

Next, we examined the role of Tip110 mutants on NF-κB activity in the context of the USP15 expression. Expression of Tip110 mutant proteins in the presence of USP15 attenuated their NF-κB basal transcriptional activity (**Figure 5D**). Interestingly, co-expression of Tip110ΔNLS or Tip110Δ274-963 mutants with USP15 led to decreases in the NF-κB activity compared to the mutants alone, indicating that USP15 expression may also affect cytoplasmic expressed Tip110 mutants. To further verify the opposing effects of Tip110 and USP15 on the NF-κB activity, we utilized NF-κB p65 protein, which is known to activate NF-κB transcriptional activity in a reporter gene assay. The results showed an enhancement in the transcriptional activation of NF-κB by the co-expression of p65 and Tip110. Expression of USP15 abrogated this enhancement effect, yet the level was still higher than Tip110 alone due to the effect of p65, indicating that p65 expression reversed the inhibitory effect of USP15 on Tip110-induced NF-κB activity (**Figure 5E**). Then, we investigated whether the abrogation of Tip110-enhanced NF-κB activity by USP15 was due to modulation of IκBα protein stability. 293T were transfected with Tip110 or USP15 alone, or in combination, then treated with TNF-α for 15 or 30 min. Similar to our previous observation, Tip110 expression led to increases in p-IκBα paralleled with decreases in IκBα protein level. While co-expression of USP15 and Tip110 reduced the IκBα protein level compared to the USP15 expressing cells only (**Figure 5F**), yet this reduction was not paralleled with increased in the p-IκBα, which is more likely due to the effect of Tip110 on the IκBα phosphorylation. Interestingly, expression of USP15 on the presence of TNF-α led to increase on IκBα phosphorylation compared to untreated cells which is not parallel with decrease on IκBα level. It has been reported that overexpression of USP15 potentiated TNF-α-induced NF-κB activation (42). In addition, deletion of USP15 N-terminal domain enhanced NF-κB activity (**Figure 5C**). Thus it’s possible that USP15 regulates NF-κB at multiple mechanisms. These data further confirming the opposing effect of Tip110 and USP15 on IκBα stability.

As we previously alluded to, Tip110 expression promoted the nuclear translocation of USP15 where both are associated (25) and the above observations prompted us to examine whether Tip110 also targeted nuclear IκBα to regulate NF-κB activity. 293T were transfected same as above then treated with leptomycin B (LMB), a nuclear export inhibitor found to sequester NF-κB/IκBα complexes in the nucleus (43), alone or together with TNF-α. Then the cells were prepared for an NF-κB reporter gene assay. As reported previously, treatment of cells with LMB inhibited NF-κB activity while further inhibition was observed by combined treatment with TNF-α (**Figure S2A**) (39, 44). However, LMB treatment had no significant effect on NF-κB activity under the context of Tip110 expression compared to untreated cells, but inhibition of NF-κB activity was observed with combined treatment with TNF-α. Furthermore, LMB treatment led to decreases in p65-induced NF-κB transcriptional activity while Tip110 co-expression resulted in a marginal increase compared to p65 expression alone (**Figure S2B**). These data indicate that USP15 expression abrogated the effect of Tip110-induced NF-κB activity and that Tip110 could regulate both nuclear and cytoplasmic IκBα protein stability.

### Tip110 Altered TNF-α-Induced NF-κB-Dependent Gene Expression

**Figure 6:**
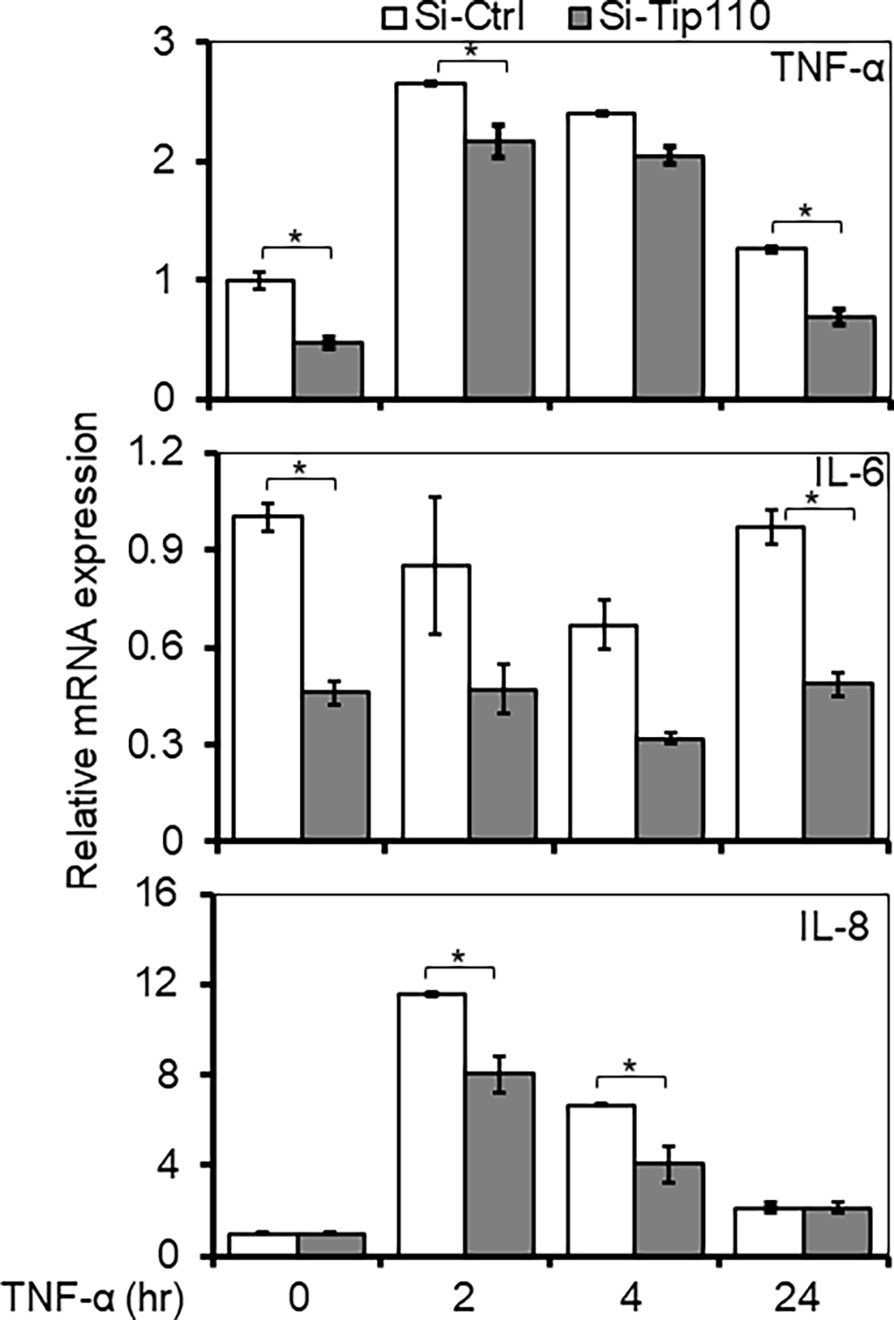
Effects of Tip110 expression on NF-κB responsive gene expression. 293T were transfected with 50 nM of Si-Ctrl or Si-Tip110, cultured for 24 hr, treated with TNF-α (10 ng/ml) for 0, 2, 4 or 24 hr, and harvested for total RNA isolation and qRT-PCR for TNF-α, IL-6, and IL-8 mRNA expression. The relative mRNA expression was normalized to β-actin and expressed as fold increases over those in Si-Ctrl-transfected and untreated cells. The data were mean ± from triplicate samples. *P < 0.05.

To gain further insight into the consequence of Tip110 effect in the TNF-α-induced NF-κB signaling pathway, we assessed the NF-κB dependent gene expression of various inflammatory cytokines when Tip110 was knocked down. 293T were transfected with control or Tip110 siRNA then stimulated with TNF-α for short (2 and 4 hr) and long (24 hr) lengths of time due to different stimulation kinetics exhibited by various cytokines in response to TNF-α treatment. The cells were harvested for the determination of mRNA level by qRT-PCR. In line with the previous findings, the overall expression of NF-κB target genes such as TNF-α and IL-6 were substantially decreased in Tip110 knockdown cells as compared to their respective control (**Figure 6**). While IL-8 expression showed no significant reduction, indicating that these differences could reflect the quantitative requirement for NF-κB by different target genes under a limited amount of Tip110 or that the regulation of IL-8 by NF-κB under the context of Tip110 acted differently than other cytokines. Recently, we reported that knockdown of Tip110 in melanoma cell line resulted in the up-regulation of IL-8 expression while no such effect was observed in the cell lines from other cancer types (22).

### Whole-Transcriptome Analysis of Tip110 Knockout Mouse Embryonic Stem Cells Revealed Dysregulation of NF-κB-Related Signaling Pathways

**Figure 7:**
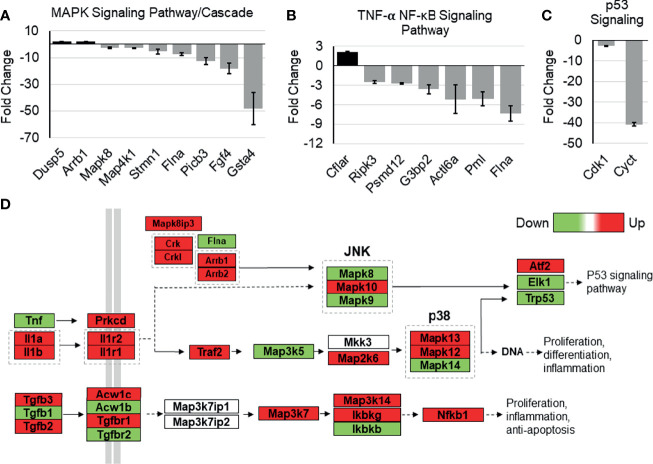
Effects of Tip110 knockout on gene expression in several NF-κB-related signaling pathways. Affymetrix Transcriptome Analysis Console software provided a list of pathways found in WIKI Pathways Beta that were most perturbed by Tip110 loss. This information was used to construct bar graphs depicting the fold change of some of the associated genes. Genes associated with MAPK **(A)**, TNF-α-NF-κB **(B)**, p53 **(C)**, signaling pathways were included. **(D)** Data from Affymetrix microarray analysis of wild-type and Tip110 knockout-mouse embryonic stem cell RNA revealed the fold changes of genes in these networks. Genes are shown in red when upregulated and in green when downregulated. Pathway information was adapted from MAPK and other signaling pathways depicted in WIKI Pathways Beta.

The possible role of Tip110 on NF-κB signaling pathway was further illustrated by an analysis of whole-transcriptome array data using Affymetrix Expression Console (TAC) and pathway analysis software that was generated previously during our investigation of the embryonic lethality instigated by knockout of Tip110 in mice (45). The microarray analysis data from the Tip110-knockout mESC revealed that several genes associated with NF-κB regulatory pathways were highly downregulated. Specifically, FGF4 (-17.8 fold) and GSTA4 (-46.7 fold) related to MAPK signaling cascades (**Figure 7A**), PMI (-4.97 fold) and FLNA (-7.26 fold) related to the TNF-α-NF-κB pathway (**Figure 7B**), CDK1 (-9.99 fold) and CYCT (-40.0 fold) related to p53 signaling pathway (**Figure 7C**), SPRY2 (-8.22 fold) related EGFR1 signaling pathway (**Figure S3A**), CDK5 (-8.4 fold) related to IL-6 signaling pathway (**Figure S3B**), and SSP1 (-39.2 fold) related to TGF-β1 receptor signaling pathway (**Figure S3C**). Interestingly, analysis of TNF-α-NF-κB, EGF, and TGF-β signaling cascades demonstrated that several genes within the cascade were also upregulated in the knockout Tip110 cells (**Figures 7D** and **S3D**), indicating there is a compensatory mechanism that was impacted these signaling pathways in the absence of Tip110, which may also explain the little effect of Tip110 on the basal level of IκBα and the gene expression of cytokines (**Figure 6**).

## Discussion

To date, there are a small number of nuclear-restricted proteins that have been reported to play a role in NF-κB signaling. However, the exact molecular mechanisms are not fully understood (46). Herein, we identified another nucleus-restricted protein, Tip110, to regulate NF-κB through a unique mechanism. Our results demonstrated that Tip110 activates NF-κB but while inhibits AP-1 activity (**Figure 1**). Nuclear localization of Tip110 was required for such activation as deletion of the NLS obliterated Tip110-induced NF-κB activity concomitant with an increase in Tip110 ubiquitination (**Figures 1**, **2**). We further investigated the potential molecular mechanisms and found that Tip110-associated shuttling protein, USP15, opposingly regulated NF-κB activity by targeting IκBα protein stability (**Figure 4**). Our findings were further substantiated using whole-transcriptome array data of Tip110 knockout mouse cells, which showed that several NF-κB and NF-κB-related pathways were dysregulated (**Figures 7** and **S3**).

Several Tip110-associated proteins have been identified (8). Interestingly, those Tip110-interacting partners have been found to play roles in NF-κB signaling through their interaction with and/or regulation of the signaling components of the NF-κB pathway (9, 10, 21, 24, 26, 27). We previously have shown that Tip110 bound to and enhanced Tat-mediated HIV-1 gene expression (28). It has been reported that HIV-1 Tat protein activates the NF-κB pathway *via* physical interaction with IκBα and p65 proteins (29). Persistent activation of NF-κB that occurs in HIV-1-infected cells results in the enhancement of the expression of proinflammatory cytokines and chemokines, which causes immune and neuron dysfunction due to chronic inflammation (47, 48). Furthermore, we also reported that Tip110 regulated several oncogenic proteins such as Y-box-binding protein 1 (YB-1), C-Myc, p53, USP15, and USP4 (9, 10, 21, 24) which regulate NF-κB signaling (49–54). These observations suggest a highly possible role of Tip110 in HIV-1-mediated chronic inflammation and cancer-associated inflammation *via* modulation of the NF-κB pathway.

Nuclear localization of Tip110 is required for its function on the NF-κB activity. Interestingly, the cytoplasmic expressed-Tip110 mutant (Tip110ΔNLS) showed a higher ubiquitination level compared with its nuclear wild-type (**Figure 2D**). Meanwhile, this difference in the ubiquitination had little effect on the level of the expressed proteins (**Figure 5D**). Tip110 expression promoted the translocation of several associated nuclear-cytoplasmic shuttling proteins such as USP4 (38), USP15 (25, 38), and YB-1 [(8), data not shown] from the cytoplasm to the nucleus where they co-localized. The complex structure of the NLS on Tip110 and importin 2α is involved a bipartite binding, and removal of Tip110 NLS prevents the entry of USP4 or USP15 in the nucleus and abrogates their subsequent deubiquitinase activity (12, 55), which may explain the detection of a higher ubiquitinated form of cytoplasmic expressed Tip110ΔNLS mutant (**Figures 2D**, **4C, E** **)**. Therefore, the NLS domain on Tip110 may play an important role in its ubiquitination and function, which merits further investigations.

Newly synthesized IκBα is accumulated in the cytoplasm but is also present in the nucleus, where it terminates NF-κB-dependent transcription (56). However, there is a requirement for the transcription of essential NF-κB-dependent genes in unstimulated cells, which requires a continual proteasome-mediated breakdown of IκBα. We found that Tip110 regulated the TNF-α-induced IκBα phosphorylation and subsequently affected its stability (**Figure 4**). However, the absence of Tip110 effect in basal level of IκBα protein (on the absence of TNF-α treatment) is likely due to the compensatory response caused by Tip110 expression as shown in **Figure 7** and **Figure S3**. Due to that, we performed CHX chase assay to determine the exogenous IκBα half-life (**Figures 4C–F**). Ectopic expression of Tip110 in the absence of TNF-α stimulation enhanced the IκBα degradation as shown by the CHX chase assay while the cytoplasmic-expressed Tip110 mutant stabilized IκBα (**Figure 4**). No interaction had been detected between Tip110 and IκBα (data not shown), and no significant effect on the IκBα mRNA level by Tip110 was observed (**Figure 4G**). Surprisingly, we found that knockdown of Tip110 also has a similar impact on the IκBα stability by the CHX chase assay. This observation further confirms the compensatory effect of Tip110 expression level as Tip110-transgenic homozygous mice apparently impacted embryonic development similar to the knockout mice [data not shown (45)].

It is important to note that activation of NF-κB transcriptional activity is not only a consequence of IκBα degradation initiated by a signaling cascade. One important example is p65, which is known to activate NF-κB transcriptional activity; however, overexpression of p65 has also been shown to increase synthesis and stabilization of IκBα (57, 58). Although Tip110 potentiated NF-κB activation, it had little effect on the basal level of IκBα protein. Therefore, we have not excluded other mechanisms such as modulation of p65 coactivators/corepressors, p65 post-translational modification, and Tip110-associated proteins that are known to play a role in NF-κB signaling. Here, we showed that Tip110-associated protein, USP15, opposingly regulates NF-κB activity by targeting IκBα protein stability.

USP15 is an oncogenic ubiquitously expressed protein that shared amino acid sequence homology with two other deubiquitinating enzymes (DUBs), USP4 and USP11 (59). However, Tip110 was found to interact with and promote nuclear translocation of USP4 and USP15 but not USP11, and this is due to β-hairpin of the linker region of the DUSP and UBL domains of both USP4 and USP15 proteins that were associated with the HAT domain of Tip110 (12, 23, 25, 38) In addition, the binding of USP15 to Tip110 is 20-fold stronger than USP4 (55). Interestingly, USP15 expression only led to Tip110 deubiquitination (25, 38). USP4 was found to inhibit TNF-α-induced NF-κB activation by targeting several upstream components of NF-κB signaling (26, 60–62). While IκBα acts as a substrate for USP15 and USP11, and this resulted in IκBα stabilization and inhibited NF-κB activation (27, 30, 63). Overexpression of USP15 inhibited Sendai Virus-induced activation of NF-κB by modulation of NF-κB phosphorylation (64). On the other hand, Zhou et al. showed that USP15 acts as positive regulator in TNF-α- and IL-1β-induced NF-κB activation through differential stabilization of TAB2/3 (42). Those observations prompted us to investigate whether Tip110-USP15 complex regulate NF-κB activity. We showed that USP15 expression obliterated Tip110-induced NF-κB activation (**Figures 5B, C**) and overexpression of Tip110 enhanced USP15-inhibited NF-κB activity (**Figures 5D, E**). Those observations were further confirmed under the context of NF-κB p65 which is known to activate NF-κB gene expression (65) (**Figure 5E**). The opposing effect of Tip110 and USP15 was a result of IκBα protein destabilization (**Figure 5F**). Unexpectedly, we observed stabilization of nuclear targeted IκBα under the context of Tip110 overexpression (**Figures 4E, F**) more likely as a result of the translocation of USP15 to the nucleus and promoted nuclear IκBα stability. Therefore, it is possible that under the pathophysiological condition, when the Tip110 level is elevated, USP15 is translocated from the cytoplasm to the nucleus and associated with Tip110. This promoted IκBα protein phosphorylation, degradation, and ultimately NF-κB activation.

It has been reported that pretreatment of cells with Leptomycin B (LMB), protein export inhibitor, inhibited NF-κB-dependent transcriptional activation mediated by TNF-α through the accumulation of IκBα in the nucleus where it became resistant to signal-induced degradation (66). We showed that LMB treatment had no effect on Tip110-induced NF-κB activity; however, in the presence of both TNF-α and LMB, Tip110 expression led to a reduction in the NF-κB activity but remained higher than transfected control (**Figure S2A**). Furthermore, synergetic activation of NF-κB was found by the co-expression of Tip110 and p65, and the addition of LMP led to a reduction in the NF-κB activity (**Figures 5E**, **S2A**). LMB treatment led to the rapid nuclear accumulation of IκBα and slower nuclear accumulation of p65 (67). Moreover, IκBα but not IκBβ is sensitive to LMB treatment in the nucleus (68). Therefore, Tip110 expression may affect IκBα protein in both cytoplasm and nucleus to regulate NF-κB activation since the localization of the p65 population is carefully controlled, presumably by the IκBα (56, 69). Thus, we speculated that other factors might also contribute to regulating IκBα stability by Tip110 in the nucleus.

Heterogeneous nuclear ribonucleoprotein A1 (hnRNPA1) is a nuclear protein that shuttles between the nucleus and the cytoplasm due to unknown conditions (70). Using proteomic study, we have shown that hnRNPA1 complexed with Tip110 (24). HnRNPA1 contributed to the control of NF-κB-dependent transcription through direct interaction and potentiation of IκBα degradation. Both nuclear and cytoplasmic expressed-hnRNPA1 proteins were able to bind with IκBα, but the nuclear hnRNAPA1 has shown a higher NF-κB transcriptional activity (71). Although the mechanism has not been investigated, the authors suggested that hnRNPA1 may act as a scaffold-like molecule, bringing IκBα into the optimal environment for signal induced-modification (72). Therefore, it is highly possible that Tip110/hnRNPA1/USP15 complex contributes to IκBα stability and NF-κB activation in the nucleus.

Recently, we reported the lethal effects of complete loss of Tip110 on mouse embryonic development. The whole-genome analysis data from knockout Tip110 embryonic stem cells compared to the wild-type (45) was utilized to elucidate the possible role of Tip110 knockout in the NF-κB pathway. The data showed that Tip110 knockout cells caused dysregulation of genes associated with NF-κB and NF-κB-related signaling pathways, including TNF-α-NF-κB, MAPK, p53, and IL-6 signaling (**Figures 7A–C** and **S3B**). Intriguingly, several genes in the EGF, TNF-α, and TGFβ receptor signaling cascade were found to be upregulated (**Figure 7D**). In the NF-κB pathway, TNF-α was down-regulated, while IL-1 cytokines and their receptors were upregulated. MAP3K5 and MAPK14 were downregulated while MAP2K6, MAPK13, and MAPK12 were upregulated. Also, TGFβ1 was downregulated while TGFβ2 and TGFβ3 were upregulated. IKKγ and NF-κB1 were upregulated, while IKKβ was down-regulated. The microarray data also showed that knockout of Tip110 cells caused dysregulation of several genes associated with apoptosis, mRNA processing, and proteasomal degradation (45). Therefore, loss of Tip110 may evoke a compensatory mechanism that may lead to an up-regulation of other proteins associated with signaling pathways at the transcriptional and/or translational level, strongly suggesting the important role of Tip110 expression in an inflammatory response. In addition, this compensatory mechanism has also been reported on the spliceosome recycling defect exerted by knockout of Tip110 in zebrafish (73).

In summary, the identification of nucleus-restricted protein Tip110 as a regulator of the NF-κB pathway unveils novel and exciting layers of regulatory specificity for NF-κB in the nucleus. In addition, our findings elucidate the important role of Tip110 expression in inflammatory diseases and cancer-related inflammation.

## Methods

### Cell Culture and Transfection

HEK 293T cell line was purchased from American Tissue Culture Collection (ATCC) and cultured in Dulbecco’s modified Eagle’s medium (DMEM) containing 10% fetal bovine serum (FBS). Mouse embryonic fibroblasts (MEF) isolation was reported previously (25). Briefly, MEF cells were isolated from embryos of 13.5-14.5 days post coitus pregnant mice and cultured in DMEM with 10% FBS and 50 μM 2-β mercaptoethanol. All media contained 100 IU/ml penicillin, 100 μg/ml streptomycin. Plasmid DNA transfection was performed using the standard calcium phosphate precipitation method for 293T. MEF cells were transfected using Lipofectamine LTX (Invitrogen) according to the manufacturer’s instructions. Si-RNA transfection was performed using Lipofectamine 2000 according to the manufacturer’s instructions (Invitrogen).

### DNA Plasmids and siRNA

Tip110.His, Tip110ΔN.His, Tip110ΔC.His, GFP-Tip110ΔNLS, Tip110ΔNLS.His, and GFP-Tip110 plasmids were described elsewhere (28, 74). Tip110Δ274-963.His, Tip110Δ387-963.His, Tip110Δ557-963.His, and Tip110Δ786-963.His plasmids were constructed in the backbone of pcDNA3 (Invitrogen) using standard PCR techniques with Tip110.His as a template and using EcoRI and XhoI cloning sites. USP15.Myc (1–952 aa), USP15ΔC.Myc (1–384 aa) and USP15ΔN.Myc (385-952 aa) were described elsewhere (25). NF-κB p65 was a kindly provided by Dr. Michael Klemsz of Indiana University School of Medicine. pNF-κB-luc and pAP-1-Luc were purchased from Clontech Laboratories Inc., CA. UB.HA plasmid was kindly provided by Dr. Mark Hannink of the University of Missouri. IκBα.HA plasmid was kindly provided by Dr. Michael Karin of the University of California (75). The IκBα-Nuc.Myc plasmid was constructed using IκBα.HA as a template, backbone of pCMV-Nuc-Myc (Addgene) and using SalI and NotI cloning sites. On-TARGETplus Tip110 siRNA (L-013447-01) and SiRNA control (D-001810-01) were purchased from Dharmacon.

### Reporter Gene Assays

The firefly luciferase activity was measured using the luciferase assay substrate (Promega, Madison, WI) according to the manufacturer’s instructions. Briefly, cells were washed with ice-cold PBS and lysed with 120 μl 1× firefly luciferase lysis buffer (Promega) at room temperature for 15 min. The lysates were centrifuged at 12,000 × *g* for 2 min to remove cell debris. The cleared lysates (5 μl) were then mixed with 20 μl firefly luciferase substrate (Promega), and the luciferase activity was measured using an Opticomp luminometer (MGM Instruments, Hamden, CT). The same cell lysates were then used for immunoblotting to confirm the protein expression.

### Immunoblot and Immunoprecipitation Analysis

Cells were washed in cold PBS and lysed in lysis buffer (50 mM Tris.HCl, pH 8.0, 280 mM NaCl, 0.5% NP-40, 0.2 mM EDTA, 2 mM EGTA, 10% glycerol, 2 mM PMSF and protease inhibitor cocktail). Lysates were cleared of cell debris by centrifugation at 12,000 *x g* and fractionated on SDS-PAGE, followed by Western blotting. For detection of Tip110 and its mutants, we used polyclonal and monoclonal Tip110 antibodies (9, 28) because not all of the Tip110 mutants were able to be recognized by the same antibody. Also, not all the constructed mutants in our study were tagged. Anti-His (abm), anti-HA (Santa Cruz Biotechnology; sc-7392), anti-p65 (Abcam; ab-7970), anti-Myc (Santa Cruz Biotechnology; sc-9E10), anti-USP15 (Santa Cruz Biotechnology; sc-100629), and anti-actin (Sigma A1978). For immunoprecipitation, 1 mg of cell lysates were incubated with 1 μg of primary antibody at 4°C on a rotating device overnight, and protein A agarose beads (Upstate) were then added for 45 min. Agarose beads were recovered and washed thoroughly three times with the above lysis buffer and processed for SDS-PAGE and Western blotting analysis.

### Immunostaining

Cells were transfected on 24-well culture plates, fixed with 4% paraformaldehyde for 20 min, and then permeabilized with 0.5% Triton X-100 for 10 min. After extensive washing with PBS, the cells were blocked with 3% BSA and incubated with anti-p65 and anti-Tip110 antibodies (1:500) for 1 hr at room temperature in a humidified chamber. Then the cells were washed and incubated with either anti-Alexa Fluor 555 or anti-Alexa Fluor 488 secondary antibodies (Invitrogen; 1:500) for 1 hr and then 1 ng/ml 4,6-diamidino-2-phenylindole (DAPI) for 5 min to stain the nuclei. The coverslips were washed with PBS and mounted with fluoromount-G (SouthernBiotech) on glass slides. Fluorescence micrographs were taken using a Zeiss Model Axiovert 200 M microscope.

### Quantitative RT-PCR Analysis of Gene Expression

RNA was extracted from cells using TRizol (Invitrogen) according to the manufacturer’s instructions. RNA (1 μg) was converted to cDNA using the iScript cDNA synthesis kit (Bio-Rad, Hercules, CA) and used as the template for PCR using SsoAdvanced SYBR green Supermix and the CFX96 real-time PCR detection system (Bio-Rad). The quantitative reverse transcription-PCR (qRT-PCR) primers used and their sequences are as follows: for β-actin, 5′-AAA CTG GAA CGG TGA AGG TG-3′ and 5′-AGA GAA GTG GGG TGG CTT TT-3′; for TNF-α, 5′-TCT TCT CGA ACC CCG AGT GA-3′ and 5-CCT CTG ATG GCA CCA CCA G-3′; for IL-6, 5′-ACA ACA AAT TCG GTA GAT CCT CG-3′ and 5′-AGC CAT CTT TGG AAG GTT CAG G-3′; for IL-8, 5′-TGC CAA GGA GTG CTA AAG-3′ and 5′-TCT CAG CCC TCT TCA AAA-3′; and, for IκBα, 5′- ACC AAC TAC AAT GGC CAC AC-3′ and 5′- ATT ACA GGG CTC CTG AGC AT-3′. Threshold cycle (*C_T_*) values were calculated using Bio-Rad CFX manager software. The 2^−ΔΔC^ *^T^* value was calculated to represent the fold change of the target gene mRNA compared to untreated siRNA control and normalized using β-actin as the reference.

### Cycloheximide Chase Assay

Cells were treated with 20 µg/ml cycloheximide (Sigma), and then the whole-cell lysates were prepared after different durations. The lysates were subjected to Western blotting analysis to identify the IκBα turnover rate alone or in the presence of Tip110 and Tip110ΔNLS. The amount of the remaining proteins was quantified by using densitometry and normalized to the β-actin.

### Data Analysis

Where appropriate, values are expressed as means ± standard deviations (SD) from triplicate samples. All comparisons were made based on the control using a two-tailed Student’s *t*-test and two-way analysis of variance (ANOVA) as appropriate. *P* values of <0.05 were considered statistically significant, <0.01 highly significant, and <0.001 strongly significant. If statistical significance (P <0.05) was determined by ANOVA, the data were further analyzed by Turkey’s *post hoc* test for multigroup Comparisons.

## Data Availability Statement

The original contributions presented in the study are included in the article/**Supplementary Material**. Further inquiries can be directed to the corresponding author.

## Author Contributions

KT: Conceptualized and executed the study, interpreted the data, and wrote the MS. SR: Helped in executing the experiments and editing the MS. AW: Analyzed whole-transcriptome array data, prepared related figures, and helped in editing the MS. YL: shared reagents and gave valuable inputs. JH: conceptualized the study, supervised experiments, supported the study, and contributed to writing the MS. All the authors approved the submission of the manuscript.

## Conflict of Interest

The authors declare that the research was conducted in the absence of any commercial or financial relationships that could be construed as a potential conflict of interest.

## Publisher’s Note

All claims expressed in this article are solely those of the authors and do not necessarily represent those of their affiliated organizations, or those of the publisher, the editors and the reviewers. Any product that may be evaluated in this article, or claim that may be made by its manufacturer, is not guaranteed or endorsed by the publisher.
