# Supplementary figures and images for: Tip110/SART3-Mediated Regulation of NF-κB Activity by Targeting IκBα Stability Through USP15
Source: Front Oncol. 2022 Apr 21;12:843157. doi: 10.3389/fonc.2022.843157 (PMC9070983; doi:10.3389/fonc.2022.843157)

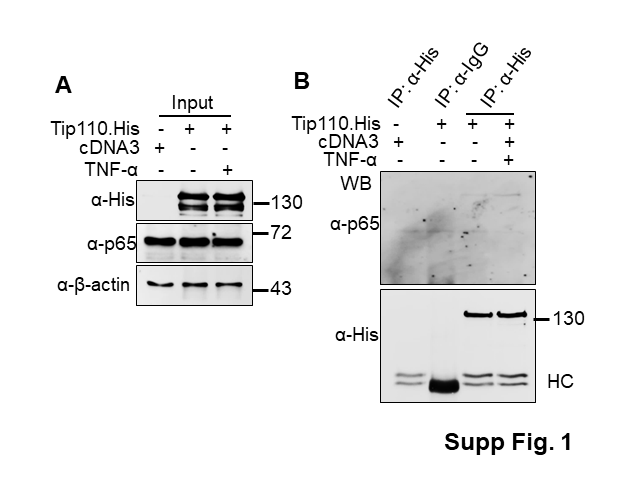

Supplement: Supplementary file 1 [file Image_1.tif]

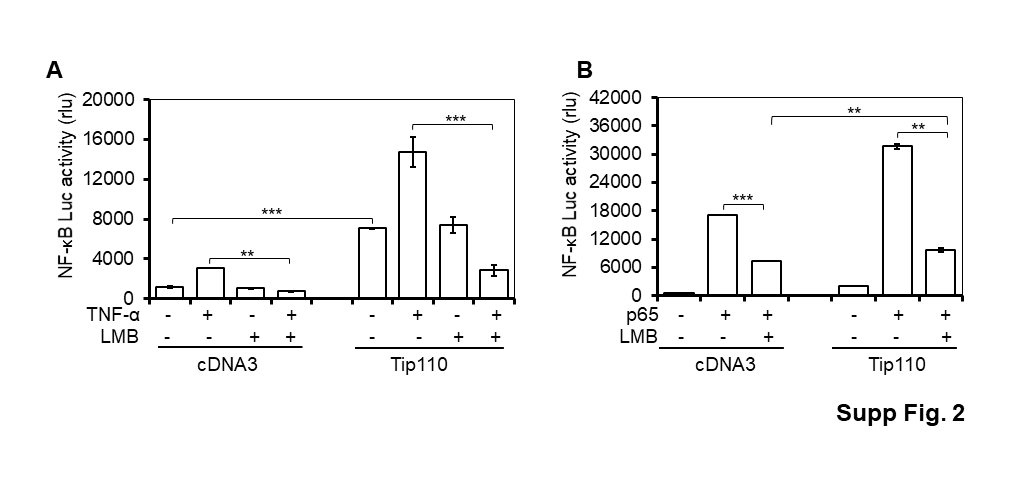

Supplement: Supplementary file 2 [file Image_2.tif]

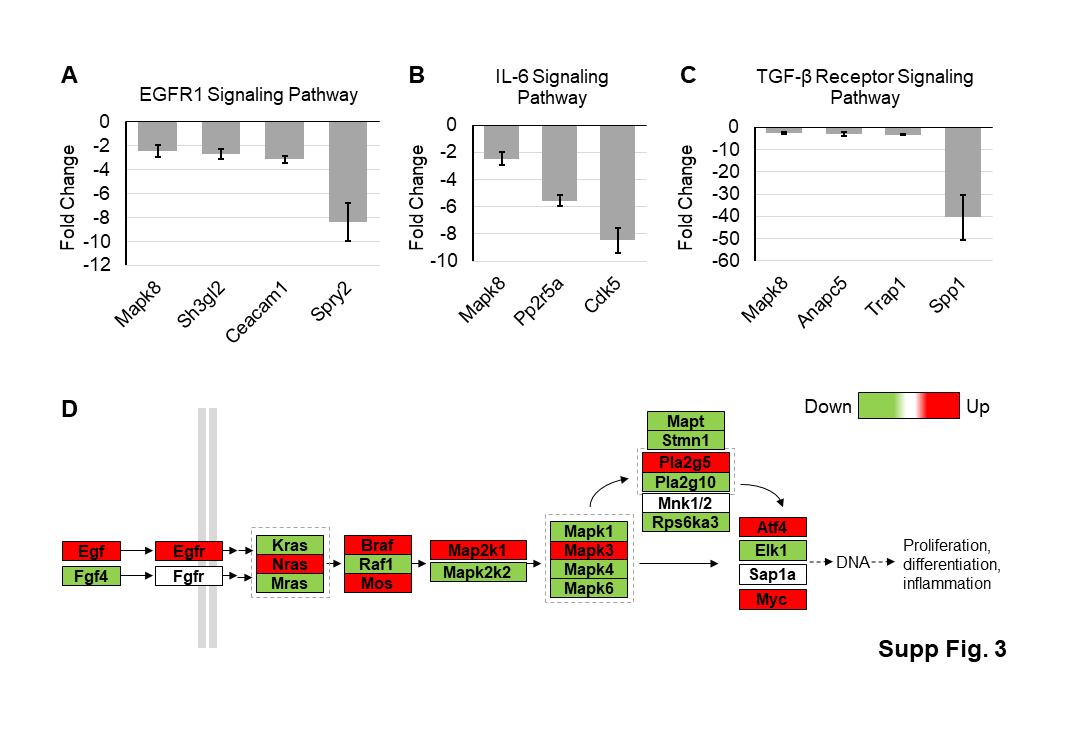

Supplement: Supplementary file 3 [file Image_3.tif]
